# Supplementary material for: Lessons learned from cross-sectoral collaboration to protect migrant farmworkers during COVID-19 in Spain
Source: PLoS One. 2025 Jan 3;20(1):e0307578. doi: 10.1371/journal.pone.0307578 (PMC11698522; doi:10.1371/journal.pone.0307578)
Supplement: Attachment — Submitted filename: renamed_4dfc2.docx [file pone.0307578.s001.docx]

**Response letter**

**PONE-D-24-02040**

**Lessons learned from institutional responses to protect Migrant Farmworkers during COVID-19 in Spain**

Dear Editor of PlosOne,

We appreciate the comments given by the editor and the reviewers, which we think sharpens the manuscript on several important points. Please, find below an explanation of how we have addressed the comments in the response letter. Changes in the manuscript have been marked using the track changes function.

Kind regards,

**Iratxe Pérez-Urdiales**

Corresponding author

- Format of the comments:

**Reviewers’ comment in bold**

Author comments in normal typeface

*Excerpts from manuscript italicized and indented*

**When submitting your revision, we need you to address these additional requirements.**

**1. Please ensure that your manuscript meets PLOS ONE's style requirements, including those for file naming. The PLOS ONE style templates can be found at**

**https://journals.plos.org/plosone/s/file?id=wjVg/PLOSOne_formatting_sample_main_body.pdf and**

**https://journals.plos.org/plosone/s/file?id=ba62/PLOSOne_formatting_sample_title_authors_affiliations.pdf**

The format of the text has been revised to comply all the requirements included in the templates

**2. We note that the grant information you provided in the ‘Funding Information’ and ‘Financial Disclosure’ sections do not match.**

**When you resubmit, please ensure that you provide the correct grant numbers for the awards you received for your study in the ‘Funding Information’ section.**

Thanks for noticing. Funding information has been changed in all the sections

**3. Thank you for stating the following financial disclosure:**

**"This work was supported by the Instituto de Salud Carlos III (ISCIII), under the grant: PI20/01310, and co-funded by the European Union"**

**Please state what role the funders took in the study. If the funders had no role, please state: ""The funders had no role in study design, data collection and analysis, decision to publish, or preparation of the manuscript."" If this statement is not correct you must amend it as needed.**

**Please include this amended Role of Funder statement in your cover letter; we will change the online submission form on your behalf.**

The statement has been included in the cover letter and the cover letter has been resubmitted

**4. Thank you for stating the following in the Acknowledgments Section of your manuscript:**

**"Acknowledgments**

**The authors thank the participants in this study who kindly shared their experiences and to the organizations that have funded the project"**

**Please be informed that funding information should not appear in the Acknowledgments section or other areas of your manuscript. We will only publish funding information present in the Funding Statement section of the online submission form.**

**Please remove any funding-related text from the manuscript and let us know how you would like to update your Funding Statement. Currently, your Funding Statement reads as follows:**

**"This work was supported by the Instituto de Salud Carlos III (ISCIII), under the grant: PI20/01310, and co-funded by the European Union"**

**Please include your amended statements within your cover letter; we will change the online submission form on your behalf.**

No funding information appears in the Acknowledgment section. And funding information has been amended in the platform and including a new statement in the cover letter.

**5. We note that you have indicated that there are restrictions to data sharing for this study. For studies involving human research participant data or other sensitive data, we encourage authors to share de-identified or anonymized data. However, when data cannot be publicly shared for ethical reasons, we allow authors to make their data sets available upon request. For information on unacceptable data access restrictions, please see http://journals.plos.org/plosone/s/data-availability#loc-unacceptable-data-access-restrictions.**

**Before we proceed with your manuscript, please address the following prompts:**

**a) If there are ethical or legal restrictions on sharing a de-identified data set, please explain them in detail (e.g., data contain potentially identifying or sensitive patient information, data are owned by a third-party organization, etc.) and who has imposed them (e.g., a Research Ethics Committee or Institutional Review Board, etc.). Please also provide contact information for a data access committee, ethics committee, or other institutional body to which data requests may be sent.**

**b) If there are no restrictions, please upload the minimal anonymized data set necessary to replicate your study findings to a stable, public repository and provide us with the relevant URLs, DOIs, or accession numbers. Please see http://www.bmj.com/content/340/bmj.c181.long for guidelines on how to de-identify and prepare clinical data for publication. For a list of recommended repositories, please see https://journals.plos.org/plosone/s/recommended-repositories. You also have the option of uploading the data as Supporting Information files, but we would recommend depositing data directly to a data repository if possible.**

**Please update your Data Availability statement in the submission form accordingly.**

**Reviewers' comments:**

5. Review Comments to the Author

**Reviewer #1: This is an interesting study focused on exploring experiences shared by social and health professionals during the COVID-19 pandemic in four specific regions of Spain: Catalonia, La Rioja, Murcia and Andalusia.**

**Summary: adequate, the methodological design needs to be introduced.**

Thanks for the appreciation. The methodological design has been included in the abstract: (line 36-44)

*This descriptive qualitative study was based on 87 personal interviews with health and social professionals from sectors such as NGOs, social services, trade unions, local institutions, and health services to promote compliance with these measures in four Spanish regions. It explored the difficulties faced by migrant agricultural workers in complying with prevention measures during the COVID-19 pandemic, the measures taken by organizations and public institutions and the health consequences of the pandemic on this group. After a reflexive thematic analysis, the results show…*

**Title: informative, does the term institutional responses encompass organizations such as NGOs?**

Thank you for pointing out this remark. Yes, we were referring to both social and institutional (governmental) entities, so to make the title more inclusive, we modified it as follows:

*“Lessons learned from cross-sectoral collaboration to protect Migrant Farmworkers during COVID-19 in Spain”*

**Introduction: broad and well presented. Authors describe the study problem well. Well justified and contextualized study.**

The words of the reviewers are highly appreciated

**Methods: a qualitative descriptive study is an appropriate design for this work. Justify why the study was carried out in those 4 regions and not in others, would the conditions have changed? This may be a limitation of the study.**

These regions were selected because they are the ones hosting the highest numbers of Migrant Farmworkers, in comparison with other regions in Spain.

It has been specified like this at the end of the introduction sections: (line 169-170)

*…in four Spanish regions that host the high numbers of MSWs (Catalonia, La Rioja, Murcia, and Andalusia)*

**What were the sample selection criteria? Although grouped sociodemographic data are given, inclusion/exclusion criteria are not clear. Define members of the sample well. Who are Members of associations/activists? Who are social integrators? Participation of all these groups in the sample should be better justified by explaining their functions in reference to these migrant workers. Is it purposive or convenience sampling?**

Thank you for pointing out this missing or confusing information. First, we have added a new sentence in the methods section explaining that the sampling strategy was purposive (since participants were selected based on their capacity to provide valuable information about the study objectives) and the inclusion criteria (health and social professionals whose tasks involved assisting migrant agricultural workers, for at least 6 months). (line 191-198)

*The interviews involved professionals from public and social entities assisting MAWs: NGO staff, health workers, social workers and educators, intercultural mediators, agricultural trade unions, and people with managerial positions in public institutions (Table 1). Participants were selected by purposive sampling and met the following inclusion criteria: professionals working in public and social entities in the four above mentioned regions, whose professional tasks included assisting MAWs, and had a minimum of six months' experience working with them.*

Second, in Table 1 we unified some similar profiles under generic classifications, since it’s true the activists and member of the associations were included in the NGOs, and the figure of social integrator is similar to a social worker.

We have also removed a participant because the interview was held in Madrid, out of the regions included in the study (even though the person have worked in those regions). (line 202)

***Table 1.*** *Socio-demographic characteristics of the participants (n=92)*

| **Sex** | |
| --- | --- |
| Men | 38 (41%) |
| Women | 54 (59%) |
| **Professional profile** | |
| Health professionals | 17 (18%) |
| Social workers | 24 (26%) |
| Cultural Mediators | 8 (9%) |
| Members of NGOs | 18 (19%) |
| COVID-19 Trackers | 2(2%) |
| Trade unionist/worker representatives | 14 (15%) |
| Political officers | 8 (9%) |
| Businessmen | 2 (2%) |
| **Province** | |
| Almería | 16 (17%) |
| La Rioja | 20 (22%) |
| Lleida | 24 (26%) |
| Huelva | 11 (12%) |
| Jaén | 4 (4%) |
| Murcia | 17 (18%) |
| **Age** | |
| 18-25 | 1 (1%) |
| 26-40 | 30 (33%) |
| 41-50 | 36 (39%) |
| 51-65 | 25 (27%) |

**Braum & Clark's thematic analysis phase could have been used as it was a qualitative/descriptive design. Incorporate an example of the coding process.**

We appreciate this suggestion. We have added an example of the coding process in a new figure 1. Find the fixed version on the picture below.

**Authors present approval from the Ethics Committee for Research with Medicines of the University Hospital Arnau de Vilanova-Lleida Territorial Management (CEIC-157 2459); This is a local committee, does this authorize them to obtain data in other regions of Spain?**

Having approval of the ethical committee allows gathering data under the conditions declared and approved by the committee, without restrictions related to the region of the participants.

**Who designed the interview script?**

The interview script was designed by the research team, including the topics that were of interest of the project, contrasted with literature. After its creation, it was piloted with 6 of the participants in the Andalusian region. After the piloting, the script was adjusted and completed with relevant topics that emerged during the interviews, and that were not included at the first version of the script.

**Who made the transcriptions of the interviews?**

The audios were uploaded to the software NVIVO transcription, which produced the first written version of them. However, due to several mistakes and misinterpretations of the audio record, they were reviewed then one by one by the different members of the team.

**The study does not make clear whether data saturation was reached.**

At the same time that the data collection took place, some members of the research team where codifying the texts. Only when data saturation was reached in main topics as health, healthcare access living and labor conditions… and in all the regions, the data collection stopped.

**Well-developed rigor section. Use of COREQ checklist is important in these studies.**

Following the suggestion of other reviewer, we moved the statement about the application of COREQ checklist to the rigor section. (line 227-228)

**Results: interesting, very explanatory, they broadly reflect the response to the stated objectives. The names of the categories are not very risky, it seems that the analysis was deductive.**

We agree that the titles of the themes were so descriptive, despite we followed an inductive approach. We improved the names of the themes and subthemes by rephrasing them in a more interpretative way. You can check the new names of the themes along the results section and in the table 2 (line 262)

**Table 2.** Themes, subthemes, categories and subcategories

| THEME | SUBTHEME | CATEGORY | SUBCATEGORY |
| --- | --- | --- | --- |
| 1. COVID's extraordinary experience of cross-sectoral collaboration | 1.1. Convergence and Synergy in measures taken by regional and local authorities | 1.1.1. The priority was that MSW were not dispersed and had accommodation  1.1.2. Accommodation for isolation in case of positive or close contact |  |
|  |  |  |  |
|  | 1.2. Solidarity Initiatives taken by NGOs during the pandemic: prevention, food and hygiene | 1.2.1. Health education  1.2.2. Food and hygiene |  |
|  |  |  |  |
|  | 1.3. Health system efforts to screen and vaccinate MWS | 1.3.1. Screening strategies  1.3.2. Vaccination strategies |  |
|  |  |  |  |
|  | 1.4. Lessons learned and good practices of cross-sectoral collaboration and networking |  |  |
| 2. Compliance or non-compliance of MSW with the preventive and containment measures of COVID-19? | 2.1. Challenges in practice to implement the prevention measures | 2.1.1. Maintaining the safety distance between MSW | At work  In accommodation |
|  |  | 2.1.2. Hygiene, disinfection and MSW’s use of protective face masks | At work  In settlements |
|  |  | 2.1.3. Performance of screening tests | PCR testing among MSW  Communication of PCR results to MSW  Registration of PCR results from MSW |
|  |  |  |  |
|  |  |  |  |
|  | 2.2. Grounds for MSW non-compliance with the preventive measures | 2.2.1. MSW concealed symptoms for fear of losing work time and/or being evicted from the home |  |
|  |  | 2.2.2. Cultural barriers and different conceptions of health/disease between MSW and the native population |  |
| 3. COVID exacerbated common health and healthcare problems of MWS | 3.1. Imprints on physical and mental health of MSW | 3.1.1. Physical health  3.1.2. Mental health |  |
|  |  |  |  |
|  | 3.2. The Odyssey of Care for MSW: Access and quality of healthcare | 3.2.1. Neglect of other diseases |  |
|  |  | 3.2.2.Telemedicine: increasing communication barriers and decreasing trust in the health system | Language barriers and diminished confidence  Increasing communication barriers |

**Discussion: I miss greater confrontation with results from similar studies in other European regions highlighting similarities/differences.**

Thank you for the suggestion; some key elements have been added to help contrast our findings from a European perspective. See the added text at the discussion section:

*Thus, the COVID-19 crisis has exposed, from a European perspective, the deep-rooted structural problem in the continent's agro-industry, especially in the south, where migrant labor operates under precarious conditions (45). In 2020, the European Commission issued guidelines to facilitate the mobility of workers deemed essential (54). Additionally, the New Pact on Migration and Asylum addressed migration as a key issue for the EU and offered a common framework for managing migration and asylum based on solidarity among all member states (55). These initiatives focused more on managing irregular migration and prioritizing market demands than on improving rights and safeguarding the health of migrants, specifically MFWs (46). In countries like Italy, actions were limited to the regularization of irregular migrant workers and the implementation of a “health pass” as a requirement for employment (56). This resulted in new roles and responsibilities for migrant workers, paving the way for the regularization of their status (57), but without substantial improvements in their social and working conditions in the short or medium term (56). On the other hand, Germany and Austria opted to allow the employment of migrants in agriculture and care, exempting them from travel bans, measures criticized for not ensuring adequate health protections for workers and being more oriented towards ensuring food availability for the entire population (58,59). In the UK, following the lockdown, agricultural organizations voiced concerns about the economic impact on agriculture due to the lack of labor, and the solution involved facilitating the importation of labor from countries like Romania and Bulgaria (60).*

*Shifting our attention back to the findings of this research centered around the Spanish example, one of the patterns identified in the measures implemented was job insecurity and precariousness of life linked to the irregular administrative situation.*

**Limitations of this study should be noted after the discussion.**

The Limitations section has been put after the main discussion text, as a subheading, and following Implications section.

**Conclusions. Although they respond to the objectives, authors should focus more on the specific conclusions of their study. The rest of the sentences should be included later in a section on lessons learned or implications for practice.**

We appreciate this suggestion. Following your advice, in the new version of the manuscript, we split the content previously in the conclusion between the conclusions and implications. Now it reads as follows:

***Implications***

*This study highlighted the imperative need to foster and maintain over time collaboration between different entities, including administrations, social and health professionals, NGOs, and companies, to enhance the quality of care offered to MWS. The synergy between different actors is essential in effectively addressing beneficiaries' needs and challenges, thus ensuring a more comprehensive and efficient response.*

*This scenario underscores the importance of comprehensively addressing health disparities, providing valuable learnings and lessons in a pandemic, and caring for MSW during future agricultural seasons. In addition, the need to address health inequalities from a holistic perspective, recognizing the interconnectedness of social, economic, and cultural factors, has been highlighted. In this regard, the figure of the cultural mediator emerged as a crucial actor in facilitating communication and understanding between workers and public health measures.*

And the new version of the conclusions now reads as follows:

***Conclusions***

*The study underlines the close relationship between health and care for MSW of migrant origin and the social asymmetries that generate inequality. The pandemic has played an essential role in shedding light on the contribution of MSW and their work. It also revealed their essential contribution and the systemic vulnerability that characterizes their situation, a pre-existing but previously ignored reality. Lessons learned manifest in improved coordination between companies, social services, administration, and NGOs in caring for workers and increased inspections to ensure compliance with laws and trade union agreements. These lessons are not only relevant in the context of a pandemic but also reveal the need for deeper structural transformations to ensure that food is produced sustainably.*

**Reviewer #2:**

**This is an interesting qualitative study on the perspective of professionals on the challenges posed by caring for immigrants who work in the Spanish agricultural sector. The introduction is good. Define the topic appropriately, provide background and justify the study**

Thank you for your positive comments

**Materials and Methods:
p. 5, line 129. The phrase "We conducted 87 semi-structured personal and online interviews with 93 participants" is confusing. If 87 interviews were carried out, how did the remaining 6 participate??? Or were there 93 interviews? Please clarify this and remove participants who did not participate in the interviews**

To avoid the confusion, we have rephrased the sentence this way: (line 189-192)

*We conducted 87 semi-structured personal and online interviews (80 individual and 6 in pair interviews) with 92 participants (54 women and 38 men)*

**Get the name of the analysis software correctly: it is ATLAS.ti (capital letters)**

Done. Thank you

**Line 153-154 (COREQ) should not focus on ethical issues but on rigor.**

Following your suggestion, we moved the statement about the application of COREQ checklist to the rigor section. (line 206-218)

**I suggest describing the data analysis based on the procedure described previously by one author. I suggest Braun and Clarck's reflective thematic analysis See for example: Clarke, V., & Braun, V. (2017). Thematic analysis. The journal of positive psychology, 12(3), 297-298.**

From line 213 to 227, how the procedure of the data analysis was done is explained.

In addition, we have referenced the following article to illustrate the way the analysis was done:

Braun V, Clarke V. Reflecting on reflexive thematic analysis. Qual Res Sport Exerc Health [Internet]. 2019 Aug 8;11(4):589–97. Doi:10.1080/2159676X.2019.1628806

**Results:
Please, provide a definition of the main categories before getting into the subtopic. The explanations of some subtopics can be improved. For example: Food and hygiene has a very brief explanation and development.**

A brief description of the main themes has been included (line 267-270 and 598-601)

The explanation of the category Food and Hygiene has been further developed, and now it reads like this: (line 340-343)

*NGOs provided food to the settlements and set up soup kitchens for seasonal workers. They also facilitated disinfection by providing the necessary materials such as water, masks and disinfectants in the settlements to prevent further spread of the virus.*

**The structure of Categories and subcategories is not clear. Some topics have two hierarchical levels and others have three. I suggest unifying this**

An inductive analysis of the data was applied, so we came to the present structure of the results. We think that changing this emerging structure based on the number of categories and subcategories is not appropriate.

**An initial table or concept map from ATLAS.ti would help to obtain an overview of the themes and subthemes developed**

Thank you for this suggestion. We created and added a new table 2 to help the reader to see all the themes and subthemes. You can consult it in the line 262 of the text or in the page 7 of the response letter, given as answer to Reviewer 1´s comment.

**Conclusions**

**I think the conclusions are too extensive and do not always derive from the results or respond to the objective. Perhaps some of them could be included in a subsection of implications for practice and policy.**

Following as well a previous comment of reviewer 1, in the new version of the manuscript, we split the content previously in the conclusion between conclusions and implications sections. Now it reads as follows:

***Implications***

*This study highlighted the imperative need to foster and maintain over time collaboration between different entities, including administrations, social and health professionals, NGOs, and companies, to enhance the quality of care offered to MWS. The synergy between different actors is essential in effectively addressing beneficiaries' needs and challenges, thus ensuring a more comprehensive and efficient response.*

*This scenario underscores the importance of comprehensively addressing health disparities, providing valuable learnings and lessons in a pandemic, and caring for MSW during future agricultural seasons. In addition, the need to address health inequalities from a holistic perspective, recognizing the interconnectedness of social, economic, and cultural factors, has been highlighted. In this regard, the figure of the cultural mediator emerged as a crucial actor in facilitating communication and understanding between workers and public health measures.*

And the new version of the conclusions now reads as follows:

***Conclusions***

*The study underlines the close relationship between health and care for MSW of migrant origin and the social asymmetries that generate inequality. The pandemic has played an essential role in shedding light on the contribution of MSW and their work. It also revealed their essential contribution and the systemic vulnerability that characterizes their situation, a pre-existing but previously ignored reality. Lessons learned manifest in improved coordination between companies, social services, administration, and NGOs in caring for workers and increased inspections to ensure compliance with laws and trade union agreements. These lessons are not only relevant in the context of a pandemic but also reveal the need for deeper structural transformations to ensure that food is produced sustainably.*

**Reviewer #3:**

**The article focuses on the difficulties faced by migrant agricultural workers in complying with prevention measures during the COVID-19 pandemic and the health consequences in Spain. The research carried out, using qualitative methods and conducted 87 semi-structured personal and online interviews with 93 participants interviews with involved professionals in social and health care: NGO 132 staff, health workers, social workers and educators, intercultural mediators, agricultural trade unions, and people with positions in public institutions. Particular emphasis is given on the consequences, their working conditions and perceptions. This is a very pertinent issue and the article deals well with this.**

**General**

**The fit between, theory and empirical analysis is well developed, and aids the author(s) in his/her/their analysis, and in framing the conclusion. The main literature mentioned covers the range of the research. The manuscript is informative regarding this particular issue in Spain, while contributing in relevant international research and literature. The author(s) addresses a significant research subject and presents interesting field material. This article could be a starting point for further research.**

Thanks for the recognition words.

**Introduction**

**A clear stating and focusing of the argument is provided. It offers a robust theoretical frame.**

Please, note that the introduction section has been further completed with new data about the characteristics of the Migrants and Migrant Farmworkers who work in different regions of Spain.

**Methods**

**Methods are appropriate and the fit between theoretical discussion and methodology is well formulated.**

Small changes have been done in the Methods section, like including the inclusion criteria for the participants (line 195-198).

*Participants were selected by purposive sampling and met the following inclusion criteria: professionals working in public and social entities in the four above mentioned regions, whose professional tasks included assisting MSWs, and had a minimum of six months' experience working with them.*

**Results**

**Results are linked suitably to the other sections of the article. A well-organized and compelling discussion of the results is provided as well. The results are of interest for practice, social and migration policy and society more generally.**

In the results section, we have provided an introductory short explanation for each theme

**Conclusions**

**The conclusions are linked to the hypothesis and background characteristics incorporated into the results.**

Based on the comments given by Reviewer 1 and 2, we split the content previously in the conclusion between conclusions and implications sections. Now it reads as follows:

***Implications***

*This study highlighted the imperative need to foster and maintain over time collaboration between different entities, including administrations, social and health professionals, NGOs, and companies, to enhance the quality of care offered to MWS. The synergy between different actors is essential in effectively addressing beneficiaries' needs and challenges, thus ensuring a more comprehensive and efficient response.*

*This scenario underscores the importance of comprehensively addressing health disparities, providing valuable learnings and lessons in a pandemic, and caring for MSW during future agricultural seasons. In addition, the need to address health inequalities from a holistic perspective, recognizing the interconnectedness of social, economic, and cultural factors, has been highlighted. In this regard, the figure of the cultural mediator emerged as a crucial actor in facilitating communication and understanding between workers and public health measures.*

And the new version of the conclusions now reads as follows:

***Conclusions***

*The study underlines the close relationship between health and care for MSW of migrant origin and the social asymmetries that generate inequality. The pandemic has played an essential role in shedding light on the contribution of MSW and their work. It also revealed their essential contribution and the systemic vulnerability that characterizes their situation, a pre-existing but previously ignored reality. Lessons learned manifest in improved coordination between companies, social services, administration, and NGOs in caring for workers and increased inspections to ensure compliance with laws and trade union agreements. These lessons are not only relevant in the context of a pandemic but also reveal the need for deeper structural transformations to ensure that food is produced sustainably.*

**Language**

**The manuscript is informative and its reading enjoyable. The author(s) has/have paid attention to the clarity of expression and readability, such as sentence structure.**

We really appreciate all your positive comments, thank you very much.

**Reviewer #4: This paper examines the response of support staff, NGOs, service providers and various state actors to migrant agricultural workers in Spain during the COVID 19 pandemic. The paper employs interviews with the above participants to address the initiatives undertaken to address migrant workers health, to minimize their risk and exposure to COVID19 and the challenges in implementing these initiatives. The paper argues that the structure of the program, “irregular administrative situation” (line 599), ignorance of support workers and the recruitment model inhibited support for migrant workers during this time.**

**The paper provides a valuable framing of this issue as there is need to document the support landscape for MAWs both prior to, during and post Covid19. It provides some evidence to support its findings by including quotes from various staff and support workers in healthcare. The paper is somewhat descriptive, but its contribution to scholarly and practical knowledge of the topic would benefit by providing more content and to a lesser degree to situate the study in current knowledge of the impact of COVID 19 on this workforce. The former is particularly important to ensure its conclusions and recommendations are convincing and useful.**

**The paper would make a greater scholarly contribution by more clearly situating the experience of MAWs in migration studies or health debates, either in the introduction or conclusion, an orientation that would make the results more transferable. The precarious nature of MAWs as the paper describes, is already well documented. However, why is it important for us to know about how MAWs were supported during COVID19? What was the existing support landscape for MAW in Spain? There is less known about the latter topic and providing more clear structural description of support would greatly enhance how scholars in this field can make sense of support for temporary agricultural workers.**

Thanks for giving the possibility and the suggestions to improve the topic. Now we offer a detailed description of the existing support landscape for MAWs in Spain prior to the pandemic. We outlined the structural challenges that MAWs typically encounter, including limited access to healthcare, inadequate housing, and the complexities of navigating legal and social services, often compounded by language barriers and uncertain immigration status. The study emphasizes that these pre-existing conditions have direct implications for the health and quality of life of MAWs, making it crucial to examine how they were supported during the pandemic.

In lines 144-161:

*The existing support landscape for MSWs in Spain before the pandemic was characterized by structural challenges, including limited access to healthcare, inadequate housing, and the complexities of navigating legal and social services, often exacerbated by language barriers and uncertain immigration status. During the pandemic, both local authorities and NGOs implemented extraordinary measures to care for MSWs, including temporary housing to ensure social distancing, mass testing, and stricter labor regulations to enhance safety in the workplace. Health education initiatives were carried out by NGOs, often in collaboration with agricultural companies, to inform MSWs about prevention measures and facilitate access to healthcare services (40,44,45).*

*However, the support landscape had its challenges. The inability to stop working, poor housing conditions, and cultural and linguistic differences hampered adherence to primary and secondary prevention measures, impacting the health of seasonal workers and their access to healthcare services. Additionally, the precarious nature of MSWs' employment and housing conditions contributed to the difficulty of implementing preventive measures effectively (21,46).*

**The paper could provide a more detailed discussion of the policy and administrative context for migrant labour in Spain, including the rights and benefits they have to healthcare prior to the pandemic and working conditions for workers (for example, how workers are contracted and what they do). Also, how participants vary in their pre-existing knowledge of, and contact with MAWs. This is important given that the paper concludes that the MAW program itself inhibits workers access to support (lines 474, 588), and that ignorance of MAWs was a factor in inhibiting their support. What for example is meant by “irregular administrative situation?”. What was the existing support/service landscape for MAWs prior to COVID 19? What were the responsibilities of employer/growers and was this laid out in work-contracts?**

The political and administrative context of migrant labor in Spain is detailed, including the rights and benefits that migrant agricultural workers (MAWs) had access to before the pandemic and the working conditions under which they operated. Prior to the pandemic, MAWs already faced precarious working conditions, including limited access to healthcare, inadequate housing, and challenges navigating legal and social services, often exacerbated by linguistic barriers and uncertain immigration status. The COVID-19 pandemic intensified these inequalities, further complicating compliance with preventive measures and exposing MAWs to greater health risks. To mitigate the impact, both local authorities and NGOs implemented extraordinary measures, including temporary accommodation, mass testing, and stricter labor regulations. However, working conditions, constant mobility, poor housing, and cultural and linguistic barriers remained significant obstacles to the effective implementation of preventive measures. These pieces of the have been added to the discussion section: (lines 752-774)

*The situation of MSW in Spain, especially during the COVID-19 pandemic, has highlighted the vulnerabilities and challenges faced by these essential workers in the agricultural sector. Before the pandemic, MSW already confronted precarious working conditions, including limited access to healthcare, inadequate housing, and difficulties navigating legal and social services, often exacerbated by language barriers and uncertain immigration status (70). The COVID-19 pandemic intensified these inequalities, further complicating compliance with preventive measures and exposing MSW to more significant health risks. Local authorities and NGOs implemented extraordinary measures to mitigate the impact, such as temporary accommodation, mass testing, and stricter labor regulations. Still, working conditions, constant mobility, precarious housing, and cultural and linguistic barriers remained significant obstacles to effective preventive measures (71).*

*The political and administrative response to the situation of MSW during the pandemic has been a subject of debate. In 2018, Spain adopted a national law that significantly expanded access to healthcare for all residents, including undocumented migrants, marking a substantial shift from a more restrictive coverage system in previous years (72).However, irregular migrants continued to face challenges in accessing healthcare, underscoring the need to address legislative and administrative barriers for the effective implementation of this law. Additionally, the pandemic has highlighted the agricultural sector's dependence on MSW and the importance of ensuring their rights and well-being, not only as a matter of social justice but also for the country's food security (71).*

**The paper also provides virtually no information about the MAW population in Spain. How many workers are there on average? Where do they come from? We only learn they are from Eastern Europe and North Africa on line 588 at the end. While the paper does acknowledge its limitation in not having interviewed MAWs, this does not preclude describing this population. This is important given that ‘cultural’ issues and language are raised as barriers and identified as important areas for increased support. The paper interprets ‘cultural issues’ described by a participant into ‘hardship in their migration journeys’, (lines 485-488). The description here is however very thin. Moreover, the paper suggests that MAWs responses to COVID initiatives were informed by their prior experiences but does not really discuss who workers are and what these experiences might be. Some description of the conditions in which MAWs work and live as well as the policy context is important here because it highlights the challenging context in which service and support can be implemented in future.**

We have included descriptive data on immigrants arriving in Spain in general and MAWs in particular in the introduction section, adding up to 7 bibliographical references to support the information. Data on % of immigrants engaged in agriculture, % of immigrants compared to natives in agriculture, type of employment, and gender have been included in the introduction:

Lines 58-71:

*In 2022, 7.534.513 foreign-born persons were registered in Spain (2). Of these, around 1.5 million citizens work in the agricultural sector, where in orchards, greenhouses, nurseries and gardens, migrant workers represent 66.83% of those hired in the sector with the professional category of labourer, and 36.63% as skilled workers. Not in vain, in 2022, the arrival in Spain of 31.219 irregular immigrants was recorded, of which almost 93% of them arrived by sea and by means of precarious boats dedicated to the illegal trafficking of people. By country of origin, the profile of irregular immigrants in Spain corresponds to citizens from Morocco, Guinea, Algeria, Mali, Côte d'Ivoire, Senegal and Tunisia (3,4) and 1.258.894 regular immigrants, the main nationalities being Colombian (with 172.241 arrivals in Spain), Moroccan (113.725) and Ukrainian (91.621) (5).*

Lines 76-81:

*By origin, the usual profile of agricultural migrant workers is that of citizens of African (Maghreb) and Eastern European (Bulgaria and Romania) origin (9). This collective became, therefore, a determining factor for many crops, such as fruit trees and vineyards, and work in greenhouses, for example (10).*

*MFWs could be contracted in advance in their origin countries due to international political agreements (specially women from Morocco in the south of the country, and Latin American and East Europe workers), or in situ spontaneously (which is more common for African men). The employment contract of migrant agricultural workers is usually temporary. Even if MFW characteristics, origins and their working situations differ between regions in Spain, around 58% of them move between provinces for temporary jobs during the different harvesting seasons (11).*

Lines 113-126:

*/…/ Women MSW in southern Spain follow a general trend of feminization of migrations motivated by the renationalization of the global care chain (26). Most women arrive with contracts from their countries of origin to work in intensive agriculture. The working conditions they found upon arrival are marked by exploitation and situations of labor and sexual harassment (27). Regarding living conditions, sub-Saharan women usually live in farmhouses and greenhouses, sharing small spaces with other women in groups of 4 to 10 people. They share rooms with beds separated by curtains. MSW women of Moroccan origin, and mainly seasonal workers from Huelva, often stay in Spain, encouraged by false promises of work and residence (migrants nearby). Without resources or a support network, MSW women live poorly in settlements where sanitary conditions are minimal and often resort to prostitution in the settlement itself (28,29).*

As well, in the introduction section we have included a sentence that RD 463/2020 article 5 mentions that COVID measures for agri-food activity were national (lines 141-143):

*The agri-food sector was considered strategic per article 15 Royal Decree 463/2020, which declares a state of alarm and establishes the necessary measures to guarantee food supply throughout the national territory. (41)*

**Reviewer #5:**

**Thank you for the opportunity to review this article. On the one hand, this is an important and relevant topic well justified by the authors. On the other hand, some areas need to be explained to a global audience, and a more in-depth explanation would be appreciated.**

**Although the focus is the migrant seasonal workers' vulnerabilities during the peak of the COVID-19 pandemic, the authors choose to interview and work with other populations as NGOs, and there is no work with the MSW. It is not clear why they made this decision it seems as if others talking about MSW experiences, so why not include them?**

This study is part of a broader two-phased project. In the first phase, the professionals considered in this study were interview and other quantitative data was collected through questionnaires. In the second phase, currently ongoing, Migrant farmworkers from different nationalities are being interviewed and also quantitative data is being collected.

In the text, this idea is represented like this:

*The study is part of a larger mix-methods research project that aims to assess the impact of job insecurity and social exclusion on the health of migrant men and women working in the agricultural sector in Spain (48).*

**Who are these migrant seasonal workers, from where, and how does the seasonal work imply?**

We provided a detailed insight into migrant agricultural workers (MAWs) in Spain, highlighting their critical importance in the country's agricultural sector, especially during harvest seasons. These workers primarily come from regions outside of Spain, seeking employment opportunities that they do not find in their home countries. Over the years, the labor composition in Spanish agriculture has undergone significant change, with a gradual transition from local labor to a predominantly migrant workforce. This shift is due to the intensification of agriculture, labor demand, and low wages associated with harvesting and crop maintenance tasks, leading the native population to leave the agricultural sector in favor of migrant workers. (lines 70-91)

*The transformations in the sector in recent decades have led to a greater dependence on MSWs. As a result of the demands of the labor activity and the low wages in the tasks of harvesting and maintenance of crops, since the 1980s, there has been a gradual change where the native population has left the sector and has been replaced by workers of migrant origin (6). Thus, throughout the year, different agricultural regions concentrate thousands of workers in search of opportunities that they cannot find in their countries of origin (7,8). By origin, the usual profile of agricultural migrant workers is that of citizens of African (Maghreb) and Eastern European (Bulgaria and Romania) origin (9). This collective became, therefore, a determining factor for many crops, such as fruit trees and vineyards, and work in greenhouses, for example (10)*

*This change in the Spanish agricultural labor composition, characterized by the intense use of migrant workers, is directly related to labor precariousness and places these workers in a position susceptible to abuse or exploitation, especially when these workers are in an irregular situation (11,12). Over the last decades, the Administrations have been increasing measures to improve these conditions, as well as controls over employers to avoid irregularities and abuses and to ensure that their rights are complied with (13). The employment contract of migrant agricultural workers is usually temporary. Even if MFW characteristics, origins and their working situations differ between regions in Spain, around 58% of them move between provinces for temporary jobs during the different harvesting seasons (14).*

**How the authors decided where to do the interviews (Comunidad), where does the interviews where done? How did they decide to include those groups of participants and no other ones?**

As we have explained before, this study is part of a broader two-phased project, which considers in a second phase the voice of the Migrant farmworkers. In the first phase, we considered interviewing the professionals who work closely with them, as they could provide information about not only about their situation, but also about the services that are provided to them. Note that we have also included the inclusion criteria in the text (line 191-198)

*The interviews involved professionals from public and social entities assisting MAWs: NGO staff, health workers, social workers and educators, intercultural mediators, agricultural trade unions, and people with managerial positions in public institutions (Table 1). Participants were selected by purposive sampling and met the following inclusion criteria: professionals working in public and social entities in the four above mentioned regions, whose professional tasks included assisting MAWs, and had a minimum of six months' experience working with them.*

**In some parts of the results section, the discourses are about seasonal workers. It is not clear if the quotes are referring to migrants or other seasonal workers.**

The participants were asked about the situation of the migrant Farmworkers, so all the results refer to this population.

**The analysis section treats migrants as if there is only one kind of migration when there are very different SDHs for each migratory context, and there is a lack of information about different politics in each region in where the interviews were done. COVID-19 regulations and migration flows are different for each of them, and it is not reflected in the text.**

In the discourses of the participants, COVID related behaviors or tendencies of different migrant origins could not be identified. To illustrate the idea that there are tendencies of origins among the migrants who work in different regions, but that the undocumented migrant move between regions, no matter their origin, we have added this piece of text:

*By origin, the usual profile of agricultural migrant workers is that of citizens of African (Maghreb) and Eastern European (Bulgaria and Romania) origin (9)* *This collective became, therefore, a determining factor for many crops, such as fruit trees and vineyards, and work in greenhouses, for example (10).*

*This change in the Spanish agricultural labor composition, characterized by the intense use of migrant workers, is directly related to labor precariousness and places these workers in a position susceptible to abuse or exploitation, especially when these workers are in an irregular situation (11,12). Over the last decades, the Administrations have been increasing measures to improve these conditions, as well as controls over employers to avoid irregularities and abuses and to ensure that their rights are complied with (13). The employment contract of migrant agricultural workers is usually temporary. Even if MFW characteristics, origins and their working situations differ between regions in Spain, around 58% of them move between provinces for temporary jobs during the different harvesting seasons (14).*

In the introduction, references about the feminization are also done, pointing that it is a phenomenon given at the southern regions of Spain.

*Women MSW in southern Spain follow a general trend of feminization of migrations motivated by the renationalization of the global care chain (26). Most women arrive with contracts from their countries of origin to work in intensive agriculture. The working conditions they found upon arrival are marked by exploitation and situations of labor and sexual harassment (27).*

**There is an important number of subcategories this make difficult to center on the focus of the study, the main goal is diffuse.**

We appreciate this suggestion. Coinciding with other reviewers, we created and added a summary table, as you can see in the line 262.

**Codes for the quote’s interviews are not clear. For example, what does the “e” mean?**

Thank you, E was refering to Interview (Entrevista) in Spanish, as that it the way we named them for the project. We replaced E by “Interview”, for not causing confusion.

**In the conclusions section l. 657-659 the authors explain how migration policies need to be re-evaluated, but it is not clear which ones (and in where) they are referring to as there a lot of different issues along the text.**

Thank you. Despite we were referring to Spanish migration policies, we agree the sentence sounded ambiguous and, following previous comments of the reviewers, we restructured the current version of the conclusions and implications section, where this tricky sentence is not included.
